# Field margins and botanical insecticides enhance *Lablab purpureus* yield by reducing aphid pests and supporting natural enemies

**DOI:** 10.1111/jen.13023

**Published:** 2022-05-20

**Authors:** Lawrence O. Ochieng, Joshua O. Ogendo, Philip K. Bett, Jane G. Nyaanga, Erick K. Cheruiyot, Richard M. S. Mulwa, Sarah E. J. Arnold, Steven R. Belmain, Philip C. Stevenson

**Affiliations:** ^1^ Department of Crops, Horticulture and Soils Egerton University Njoro Kenya; ^2^ Department of Biological Sciences Egerton University Njoro Kenya; ^3^ Natural Resources Institute University of Greenwich Chatham Maritime UK; ^4^ Nelson Mandela African Institution of Science and Technology Arusha Tanzania; ^5^ Royal Botanic Gardens, Kew, Kew Green Richmond UK

**Keywords:** botanical insecticides, conservation biological control, field margin, integrated pest management, legume cropping systems

## Abstract

Botanical insecticides offer an environmentally benign insect pest management option for field crops with reduced impacts on natural enemies of pests and pollinators while botanically rich field margins can augment their abundance. Here, we evaluated the non‐target effects on natural enemies and pest control efficacy on bean aphids in Lablab of three neem‐ and pyrethrum‐based botanical insecticides (Pyerin75EC®, Nimbecidine® and Pyeneem 20EC®) and determine the influence of florally rich field margin vegetation on the recovery of beneficial insects after treatment. The botanical insecticides were applied at the early and late vegetative growth stages. Data were collected on aphids (abundance, damage severity and percent incidence) and natural enemy (abundance) both at pre‐spraying and post‐spraying alongside Lablab bean yield. The efficacy of botanical insecticides was similar to a synthetic pesticide control and reduced aphid abundance by 88% compared with the untreated control. However, the number of natural enemies was 34% higher in botanical insecticide‐treated plots than in plots treated with synthetic insecticide indicating that plant‐based treatments were less harmful to beneficial insects. The presence of field margin vegetation increased further the number of parasitic wasps and tachinid flies by 16% and 20%, respectively. This indicated that non‐crop habitats can enhance recovery in beneficial insect populations and that botanical insecticides integrate effectively with conservation biological control strategies. Higher grain yields of 2.55–3.04 and 2.95–3.23 t/ha were recorded for both botanical insecticide and synthetic insecticide in the presence of florally enhanced field margins in consecutive cropping seasons. Overall, these data demonstrated that commercial botanical insecticides together with florally rich field margins offer an integrated, environmentally benign and sustainable alternative to synthetic insecticides for insect pest management and increased productivity of the orphan crop legume, Lablab.

## 1 INTRODUCTION

Natural or engineered field margins in and around crops provide shelter and floral resources for natural enemies and can augment their abundance and pest regulating services (Knapp & Řezáč, 2015; Rowe et al., 2021; Skirvin et al., 2011) even at low prey density (Amaral et al., 2016; Ben‐Issa et al., 2017). Natural enemies can be further supported and conserved through more sustainable agricultural practices including the use of selective and lower doses of insecticides (Roubos et al., 2014;) and using botanical insecticides (Stevenson et al., 2017). Synthetic insecticides are reported to be acutely toxic to insect pests and natural enemies (Suma et al., 2009). Botanical insecticides, on the other hand, include a range of active ingredients extracted from plants that exhibit insecticidal or less toxic repellent and antifeedant effects, and growth and reproductive inhibitory effects (Braimah et al., 2014). In contrast to persistent synthetic insecticides, the active components in botanical insecticides degrade rapidly in nature often owing to their instability especially in UV light, and consequently, they have lower impacts on predators and parasitoids of pests (Stevenson et al., 2017). However, combining field margins and botanical insecticides requires careful assessment of their individual and combined effects on pests and natural enemies, and the overall impact on crop yield (Amoabeng et al., 2020).

Lablab (*Lablab purpureus* L.) is a versatile multipurpose food legume that could be used as a model crop to test the integration of such strategies on orphan crops, which often lack good phytosanitary support to manage pest insects (Venzon et al., 2020). Lablab green pods and leaves are used as fresh vegetables, dry seeds provide dietary proteins and the crop is also important animal fodder (Maass et al., 2010; Mondal et al., 2017) and can be used as green manure or as a cover crop (Carsky et al., 2001; Cheruiyot et al., 2011; Northup & Rao, 2015). Lablab is a drought‐tolerant crop legume (Maass et al., 2010) that is suited to cropping systems affected by increasing temperatures and drying climate and representative of a number of underutilised or orphan crops that may help mitigate the challenges of climate change. However, sustainable pest management options have not been widely studied on Lablab nor how field margin vegetation mitigates negative impacts of pesticide use or facilitates benefits towards conservation biological control. The production of Lablab is constrained by numerous insect pests including the black bean aphid (*Aphis fabae*) (Boit et al., 2018; Cork et al., 2009; Tembo et al., 2018). The black bean aphid damage causes yellowing of leaves, desiccation, stunting in older plants and sometimes death of affected plants (Mwangi et al., 2008). However, rigorous data on yield losses are not available for Lablab.

Current control strategies for aphids are dependent on the use of broad‐spectrum synthetic insecticides (Stevenson et al., 2017). Although synthetic insecticides play an important role in aphid management, their negative effects on non‐target organisms, the environment and the health of farmers and consumers continue to be a problem (Mkenda et al., 2015, 2019). Aphids have numerous natural enemies that could be conserved to replace (or minimize) the use of broad‐spectrum insecticides (Kindlmann & Dixon, 2010). Pyrethrum and neem products are well‐established commercial pesticides based on known active ingredients (pyrethrins and tetranortriterpenoids) (Chaudhary et al., 2017). The adoption of botanical insecticides is limited due to costs and variable efficacy against target pests, which can be attributed to the rapid breakdown of bio‐active compounds (Sola et al., 2014). However, with the increasing interest in sustainable pest control and reducing persistent agricultural products, there is a need to evaluate the field performance of these botanical insecticides on insect pests and to understand their impact on natural enemies on orphan crop legumes (Venzon et al., 2020). Here we have focused on the African legume Lablab (*Lablab purpureus* (L.) Sweet).

Integrated Pest Management (IPM) draws on the combination of different pest control methods to maintain pest populations below economically important thresholds and minimise non‐target effects (Amoabeng et al., 2020). Bean aphids can be controlled using natural enemies at levels that mitigate against severe losses without reliance on chemical pesticides (Bianchi et al., 2006; Bianchi & Wäckers, 2008; Rand et al., 2006). The provision of suitable refuge and additional non‐crop habitat can serve to augment natural enemy populations in small‐holder farming systems and reduce pest build‐up in the crop (Nyaanga, 2008; Ndakidemi et al., 2021; Arnold et al., 2021). The floral diversity can support higher longevity, fecundity and predation rates of natural enemies promoting higher abundance and which translate to additive levels of biological control (Charles & Paine, 2016; Pan et al., 2020). Increasing natural enemy species richness has been attributed to strengthening biological control through multiple mechanisms (Jonsson et al., 2017). In contrast, Straub et al. (2007) argued that the conservation of natural enemy species can reduce or has no effect on biological control. High natural enemy abundance favoured by increased plant diversity in and around field crops provides the predators and parasitoids with a wide array of alternative prey (nectar and pollen), which can take natural enemies away from crops and negatively affect biological control (Jonsson et al., 2017; Venzon et al., 2019)

The use of botanical insecticides alongside natural enemy conservation potentially offers an integrated and effective alternative to synthetic insecticides for pest control. Low concentrations of botanical insecticides such as neem‐based products have low negative impacts on natural enemies, which is important for the conservation of biological control (Venzon et al., 2020). Furthermore, the conservation of natural enemies can complement insecticide use by preying on or parasitizing insect pests that survive or recolonize crops after insecticide application (Snyder, 2019). Here we hypothesised that by acting as a reservoir for natural enemies, field margin vegetation could reduce pest incidence in crop fields and support a more rapid recovery of natural enemy populations after selective application of botanical insecticides compared with synthetic products. To test this hypothesis we evaluated the impacts of botanical insecticides on aphid pests and their natural enemies used in combination with florally enriched margins around Lablab.

## 2 MATERIAL AND METHODS

### 2.1 Study site

Field trials were located at the agronomy teaching and research field, Egerton University, Nakuru County Kenya (0^°^ 20' S, 35^°^ 56' E) with an altitude of 2238 m above sea level, annual precipitation of about 1200 mm and a mean annual temperature range of 17°C–22°C. Soils are well‐drained dark reddish clays, classified as Mollic Andosols, within an agriculturally high potential agro‐ecological zone, lower highland 3 (LH3) in the Kenya Highlands (Jaetzold et al., 2012). The land area was 8 Ha predominantly inhabited by weed species as it had remained uncultivated from the previous season. The field was typically used for research and the crops grown on the site varied from one season to another. The region is categorized as a high agricultural zone, hence the soils are considered to be nutrient‐rich and to support high plant species richness.

### 2.2 Experimental design and treatment applications

Field trials were carried out during May to December 2019 and March to November 2020 cropping seasons. The experimental field was disc ploughed and harrowed before plots measuring 10 m × 10 m and 10 m apart were demarcated for use during planting. The plot dimensions used were smaller than a typical field but were considered appropriate as related studies had been conducted using similar plot sizes (Hatt et al., 2017). The first treatment level was for experimental blocks to be planted in the presence of field margin vegetation or for margin vegetation to be absent (Appendix S1). Thus, 2 weeks before the bean crop was planted, field margin vegetation was sown with plant plugs to give the field margin plants time to establish. The plot margins were created with four common flowering weed species (*Bidens Pilosa* L., *Tagetes minuta* L., *Ageratum conyzoides* L. and *Galinsoga parviflora* Cav.). These species were chosen because they are annuals and occur in abundance around the farms in the region. The selection was also guided by previous studies, which indicated that these species had an effect on arthropod populations (Amoabeng et al., 2020; Quispe et al., 2017; Souza et al., 2019; Zhang et al., 2021). The seeds of each species were mixed in equal proportions (by weight) and sown around each plot, which had plant margin treatments. The margin species were planted 0.5 m from the outer row of the Lablab crop and 0.5 m width. To ensure uniform emergence of the plant species the planting area was prepared to fine tilth. After the establishment of plot margins, lablab bean variety DL‐1002 was planted at a spacing of 60 cm by 30 cm, two seeds per hill, with an equivalent of 1112 plants per plot. At planting, NPK (23:23:0) fertilizer was applied at the rate of 60 Kg N ha^‐1^ and 60 Kg P_2_O_5_ ha^‐1^.

The second treatment level involved treatments consisting of three commercially available botanical insecticides: Pyerin 75EC®, Pyeneem 20EC® (Manufacturer: Twiga Chemical Industries Limited) and Nimbecidine® (Manufacturer: T. Stanes and Company Limited) and a synthetic insecticide Duduthrin 1.75EC® (Manufacturer: Twiga Chemical Industries Limited) as a positive control and an untreated negative control. The pyrethrum‐ and neem‐based botanical insecticides were selected since they were well‐established and available in the market (Campos et al., 2019; Sola et al., 2014). The insecticides are also registered to control a wide range of insect pests including, spider mites (*Tetranycus urticae*) whiteflies (*Bemisia tabacci*) and Tomato leafminer (*Tuta absoluta*) (Stevenson et al., 2017). However, there is surprisingly little field evidence of their effects on beneficial insects and no report of their use on natural enemies of bean aphids in Lablab. The 5 insecticide treatment levels and 2 field margin treatment levels were laid out in a randomized complete block design (RCBD) with four replications per treatment combination. Many arthropods are known to be highly mobile (Sorribas et al., 2016) therefore, to minimize the movement of insects within the experimental plots, all surrounding vegetation was cleared throughout the growing season except for the border margins. Active ingredients and applied doses are described in Table 1. The application rates were followed as per the manufacturer's recommendation. The insecticides were applied twice, with the first spraying done at 42 days after planting (DAP) when the crop entered the second trifoliate and the second spraying at 70 DAP during the sixth trifoliate. These two growth stages were selected since aphids inflict severe damage at the vegetative growth stage, attacking auxiliary buds and growing points.

**TABLE 1:** Active ingredients and dose rates of botanical insecticides and synthetic insecticide (Duduthrin) used in the study

Trade name	Rate of application (L/Ha)	Active ingredients (a.i., %)	% a.i. composition	a.i. dose (L/Ha)
Pyeneem	2.5	Natural pyrethrins 1% w/v	1.00	0.025
		Neem oil 1% w/v	1.00	0.025
		Inert ingredients 98% w/v	98.00	2.450
Pyerin	2.5	Natural pyrethrins 1% w/v	1.00	0.025
		Neem oil 1% w/v	1.00	0.025
		Garlic extract 25% w/v	25.00	0.625
		Inert ingredients 73% w/v	73.00	1.825
Nimbecidine	3.0	Azadirachtin 0.03% w/v	0.03	0.0009
		Neem oil 90.57%	90.57	2.7171
		Inert ingredients 9.4% w/v	9.40	0.282
Duduthrin (+ve control)	2.0	Lambda cyhalothrin 1.75% w/v	1.75	0.035
		Inert ingredients 98.25% w/v	98.25	0.197

w/v = Weight by volume.

### 2.3 Aphid pests

Data on aphid abundance, damage severity and percent incidence were collected 1 day before spraying and 7, 14 and 21 days after spraying for the two applications across all treatments and controls. Aphid abundance measurements were obtained by visual observation and scoring numbers using an index. Due to the high reproductive rate of aphids a categorical scale was used to assess aphid abundance, 1= no aphids; 2 = a few scattered aphids (1–100); 3 = a few small colonies (101–300); 4 = several small colonies (301–600); 5 = large isolated colonies (601–1000); and 6 = large continuous colonies (>1000) (Aken et al., 2013; Mkenda et al., 2015). The data were collected from ten randomly selected plants from the inner five rows falling within the sampling area in each treatment. The severity of damage caused by aphids on Lablab was determined by visually observing and scoring the level of damage over the same assessment times and selected plants. The severity of damage was assessed using a 1–5 scale widely adopted in the literature, where; 1= no infestation or damage, 2 = light damage and infestation, <25% plant parts damaged or infested, 3 = average damage and infestation, 26%–50% plant parts damaged, 4 = high infestation and damage, 51%–75% plants parts damaged showing yellowing of lower leaves and 5 = severe infestation, >75% damage resulting to plants with high infestation levels with yellow and severely curled leaves or dead plant (Mkenda et al., 2015). The incidence of aphids was determined by visually examining and counting the number of aphids damaged/infested plants by randomly sampling 30 plants from the inner five rows in each replicate. Assessments were made over the same sampling times and expressed as percentage incidence.

### 2.4 Natural enemies

Yellow pan traps were deployed to collect NEs as these were shown to be effective at catching a range of species in Kenyan legume agricultural systems in previous work by Mwani et al. (2021). Additionally, the use of pan traps to assess populations of natural enemies has recently been undertaken effectively (Shweta & Rajmohana, 2018; Thant et al., 2016). Furthermore, pan traps can be deployed easily in the crop, catching insects throughout the deployment period whereas other approaches such as sweep netting may be biased towards daytime‐active insects and may miss small insects like parasitoid wasps and can also damage the crop. The traps were set up at the centre and the edge of each replicated plot to sample natural enemies. The traps were set at ground level and spaced at 20 m from one experimental plot to another. The pan traps were made using 20 cm diameter yellow plastic plates filled three‐quarters with water with two drops of liquid soap mixed in to help break the surface tension. Sampling was carried out twice, 1 day before and 7 days after spraying, with traps collected after 48 h. The traps were set up concurrently with the assessment of aphids. All arthropods captured in each trap were transferred into 50‐ml falcon tubes containing 75% ethanol. Arthropod samples were sorted to identify key selected families of natural enemies associated with aphids (parasitic wasps, tachinid flies, ladybird beetles), recording the number per trap.

### 2.5 Bean harvest and yield

Yield data are presented here to show the influence of field margins and the impact of botanical insecticides as compared to conventional synthetic insecticides. Grain yield and related agronomic data were collected at physiological maturity when pods turned brown. Plant height was measured from the ground level to the tip of the main stem. Above‐ground biomass from each treatment was taken from 10 plants randomly selected from the middle five rows, using destructive sampling where the selected plants were uprooted at pod set when the plants were expected to be close to the peak of dry matter accumulation. The plants were dried at 65°C in an oven for 24 h and dry weight was recorded. The number of pods per plant was counted in each plant from 10 plants randomly selected from the inner middle rows categorised as either clean or damaged. Similarly, the number of seeds per pod was determined by threshing each pod and counting the seeds. The weight of a hundred seeds was determined using an electronic digital weighing balance (maximum weighing 3 kg; Manufacturer: Comglobal Solutions). For grain yield, pods were harvested separately within the sampling area for each treatment. Pods were sun‐dried for 2 days and threshed with the moisture content recorded using a digital moisture meter (Manufacturer: Dramiński S.A.). After attaining 13% moisture content, grains from each treatment were weighed separately using a portable digital scale (maximum weighing 40 kg; Manufacturer: Comglobal Solutions) and converted to tons ha^‐1^ using the following formula:

$$\text{Grain yield}\;\left(\text{tons}\;{\mathrm{ha}}^{-1}\right)=\frac{\text{Grain weight}\;\mathrm{per}\;\text{plot}\;x\;10}{\text{Harvest area}\;\left(m^{2}\right)}$$

### 2.6 Data analysis

The data used for analysis were the mean values from each replicate. Data on percent incidence and natural enemy counts were subjected to arcsine and square root ($\sqrt{x+1}\;\;$)transformation, respectively, to correct for heterogeneity of treatment variances. Effects of cropping seasons, botanical insecticides, field margin vegetation and their interactions were subjected to Analysis of Variance (ANOVA) for aphids’ abundance, damage, severity, percent incidence, natural enemy abundance and grain yield. The sampling time and cropping seasons were regarded as repeated measures and the means comparisons were done for field margins, botanical insecticides and their interaction effect. Pearson correlation matrix was used to test the association between the response variables. The association was to test how aphid abundance influenced damage severity, incidence and natural enemies. The means of treatments and interactions were compared using the least significant difference (LSD) test at a significant level of *p* ≤ 0.05. All analyses were done using XLSTAT version 2019.2.2.59614 (Addinsoft, 2019). XLSTAT statistical and data analysis solution (Boston, MA, USA. https://www.xlstat.com).

## 3 RESULTS

### 3.1 Aphid abundance, severity and incidence

**FIGURE 1:**
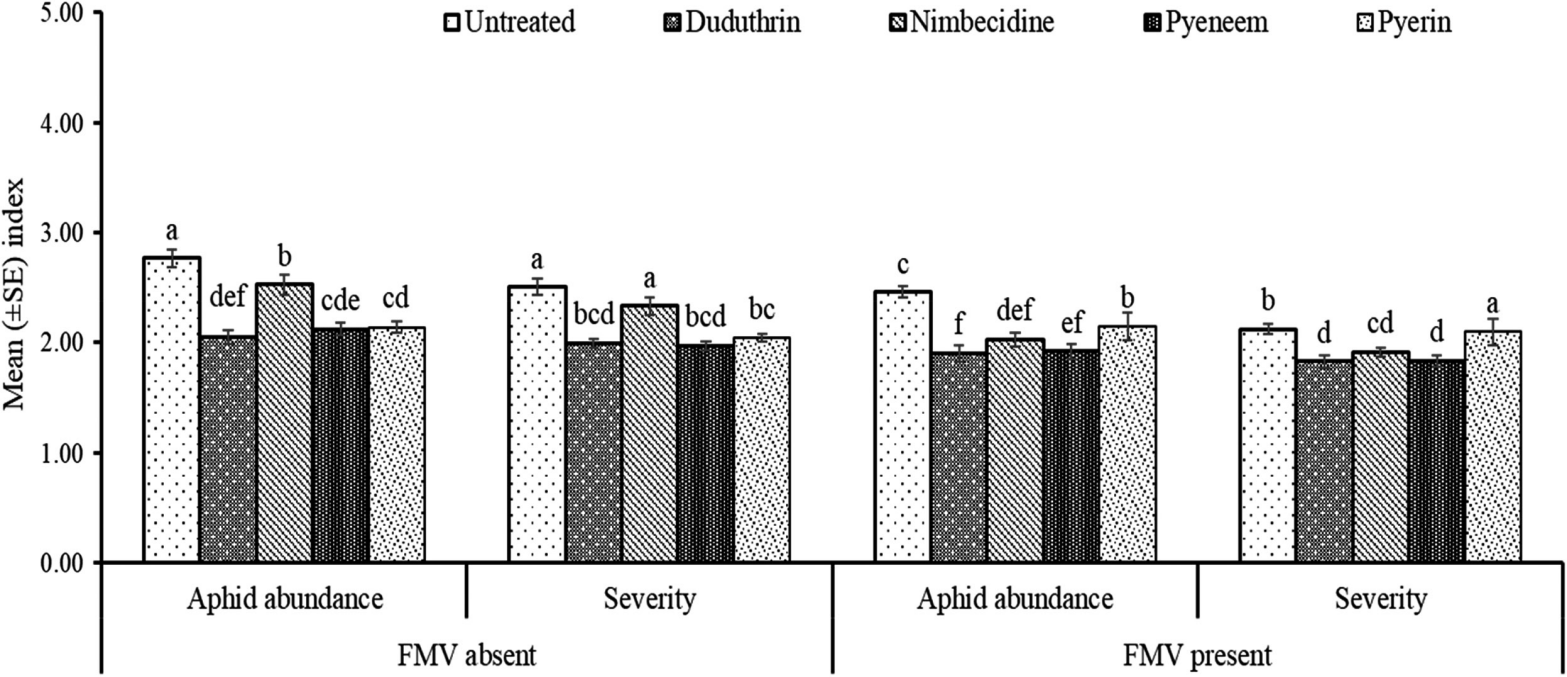
Mean (±SE) of aphid abundance and damage severity as influenced by botanical insecticides and field margin vegetation. Columns bearing the same letters are not significantly different using Fisher’s Least Significant Difference at (*P* 〈 0.05)

**FIGURE 2:**
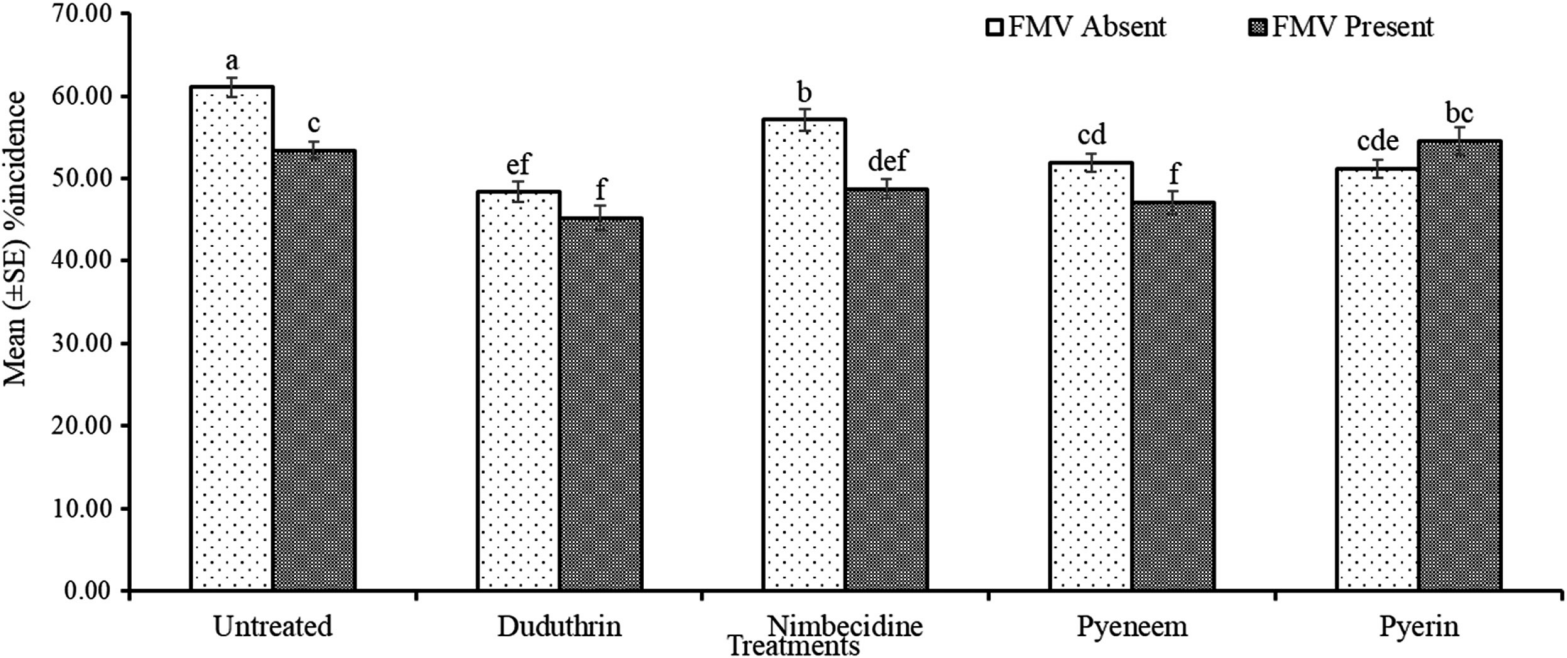
Mean (±SE) of aphid percent incidence as influenced by botanical insecticides and field margin vegetation. Columns bearing the same letters are not significantly different using Fisher’s Least Significant Difference at (*P* 〈 0.05)

The Analysis of Variance indicated interactive effects between all three parameters of season, field margin and pesticide treatment for aphid abundance, damage severity and percent incidence (Table 2). Cropping season showed some minor differences in aphid parameters but generally followed the same trends, permitting the data to be combined for the two cropping seasons (Figures 1 and 2). The botanical insecticides were able to reduce aphid numbers and damage in comparison to the untreated control and were often as good as the synthetic pesticide, Duduthrin (Figure 1). The botanical insecticides in the presence of field margin vegetation provided lower reductions in aphid abundance, severity and incidence as compared to the absence of field margins (Figures 1 and 2). The Pearson correlation analysis showed a significant (r = 0.994*** and r = 0.910***) positive association between aphid abundance and damage severity and percent incidence, respectively. A positive significant (r = 0.913***) correlation was also observed between damage severity and percent incidence.

**TABLE 2:** Analysis of variance for the aphid abundance, damage severity and percent incidence on Lablab bean for two cropping seasons (May‐December 2019 and March‐November 2020), botanical insecticides (Nimbecidine, Pyeneem and Pyerin), Duduthrin and untreated control, in the presence or absence of field margin vegetation (FMV)

Source of variation	df	Abundance	Severity	Incidence
Season	1	407.129	263.076	51.826
		<0.0001	<0.0001	<0.0001
Margin vegetation	1	30.814	24.681	32.770
		<0.0001	<0.0001	<0.0001
Treatment	4	34.842	25.901	24.037
		<0.0001	<0.0001	<0.0001
Replicate	3	4.039	4.748	4.809
		0.007	0.003	0.003
Season X Margin vegetation	1	18.824	22.904	25.639
		<0.0001	<0.0001	<0.0001
Season X Treatment	4	20.370	14.548	14.983
		<0.0001	<0.0001	<0.0001
Margin vegetation X Treatment	4	23.470	17.387	8.467
		<0.0001	<0.0001	<0.0001
Season X Margin vegetation X Treatment	4	21.784	17.234	8.433
		<0.0001	<0.0001	<0.0001
*R* ^2^		0.585	0.503	0.361
F		39.579	28.418	15.834
Pr > F		<0.0001	<0.0001	<0.0001

### 3.2 Natural enemy abundance

**FIGURE 3:**
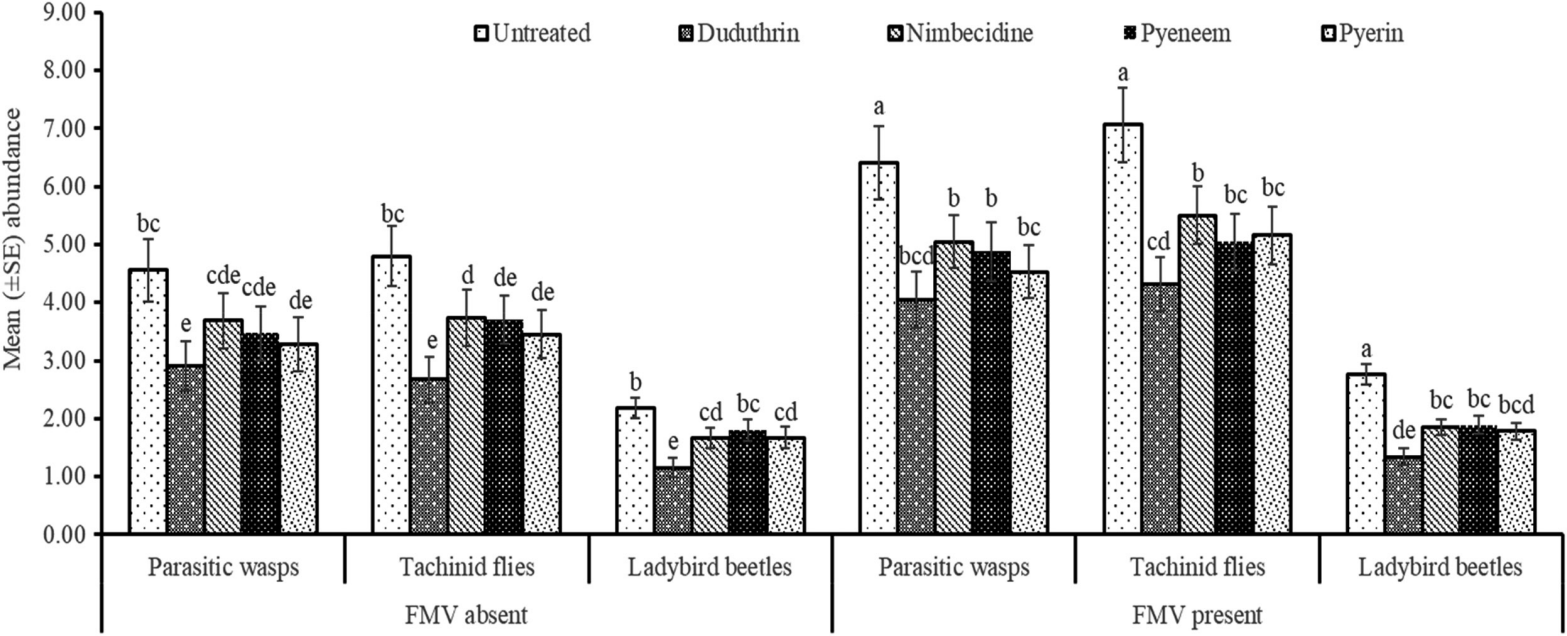
Mean abundance (±SE) of parasitic wasps, tachinid flies and ladybird beetles as influenced by botanical insecticides and field margin vegetation. Columns bearing the same letters are not significantly different using Fisher’s Least Significant Difference at (*P* 〈 0.05)

Arthropods captured in the pan traps were first grouped into the general category of aphid natural enemies comprising mainly predators and parasitoids. From the initial sorting, a total of 6808 insect natural enemies were collected during the two cropping seasons. The major groups identified were parasitic wasps (Braconidae and Ichneumonidae) 40%, tachinid flies (Tachinidae) 43% and ladybird beetles (Coccinellidae) 17%. The Analysis of Variance indicated there was only an interactive effect between season and field margin vegetation, with no significant interactions between all other parameters (Table 3). Generally, in plots with field margin vegetation, more natural enemies were collected as compared to plots with no field margin vegetation (Figure 3) The presence of field margin vegetation was particularly beneficial to parasitic wasps and tachinid flies where their numbers were nearly doubled in comparison to plots with no field margins (Figure 3). Ladybird beetle numbers were generally less affected by the presence or absence of field margin vegetation (Table 3). The botanical insecticide treatments reduced the number of natural enemies in comparison to the untreated controls; however, the reductions with the botanical insecticides were overall less detrimental compared with the synthetic pesticide Duduthrin (Figure 3). Correlation analysis revealed that there was a positive significant (r=0.638**) association between aphid abundance and natural enemy population.

**TABLE 3:** Analysis of variance for the abundance of key natural enemy species found on Lablab bean for two cropping seasons (May‐December 2019 and March‐November 2020), botanical insecticides (Nimbecidine, Pyeneem and Pyerin), Duduthrin and untreated control, in the presence or absence of field margin vegetation (FMV)

Source of variation	df	Parasitic wasps	Tachinid flies	Ladybird beetles	Overall abundance
Season	1	339.436	380.136	42.145	269.531
		<0.0001	<0.0001	<0.0001	<0.0001
Margin vegetation	1	30.852	52.186	5.233	40.620
		<0.0001	<0.0001	0.022	<0.0001
Treatment	4	7.099	10.701	15.030	12.122
		<0.0001	<0.0001	<0.0001	<0.0001
Replicate	3	0.074	0.194	0.247	0.013
		0.974	0.901	0.863	0.998
Season X Margin vegetation	1	12.928	23.695	20.933	9.344
		0.000	<0.0001	<0.0001	0.002
Season X Treatment	4	0.780	1.623	1.981	0.510
		0.538	0.167	0.096	0.729
Margin vegetation X Treatment	4	0.227	0.382	0.773	0.405
		0.923	0.822	0.543	0.805
Season XMargin vegetation X Treatment	4	0.161	0.063	1.148	0.049
		0.958	0.993	0.333	0.995
*R* ^2^		0.403	0.451	0.190	0.376
F		18.932	23.076	6.581	16.904
Pr > F		<0.0001	<0.0001	<0.0001	<0.0001

### 3.3 Lablab harvest yield

**FIGURE 4:**
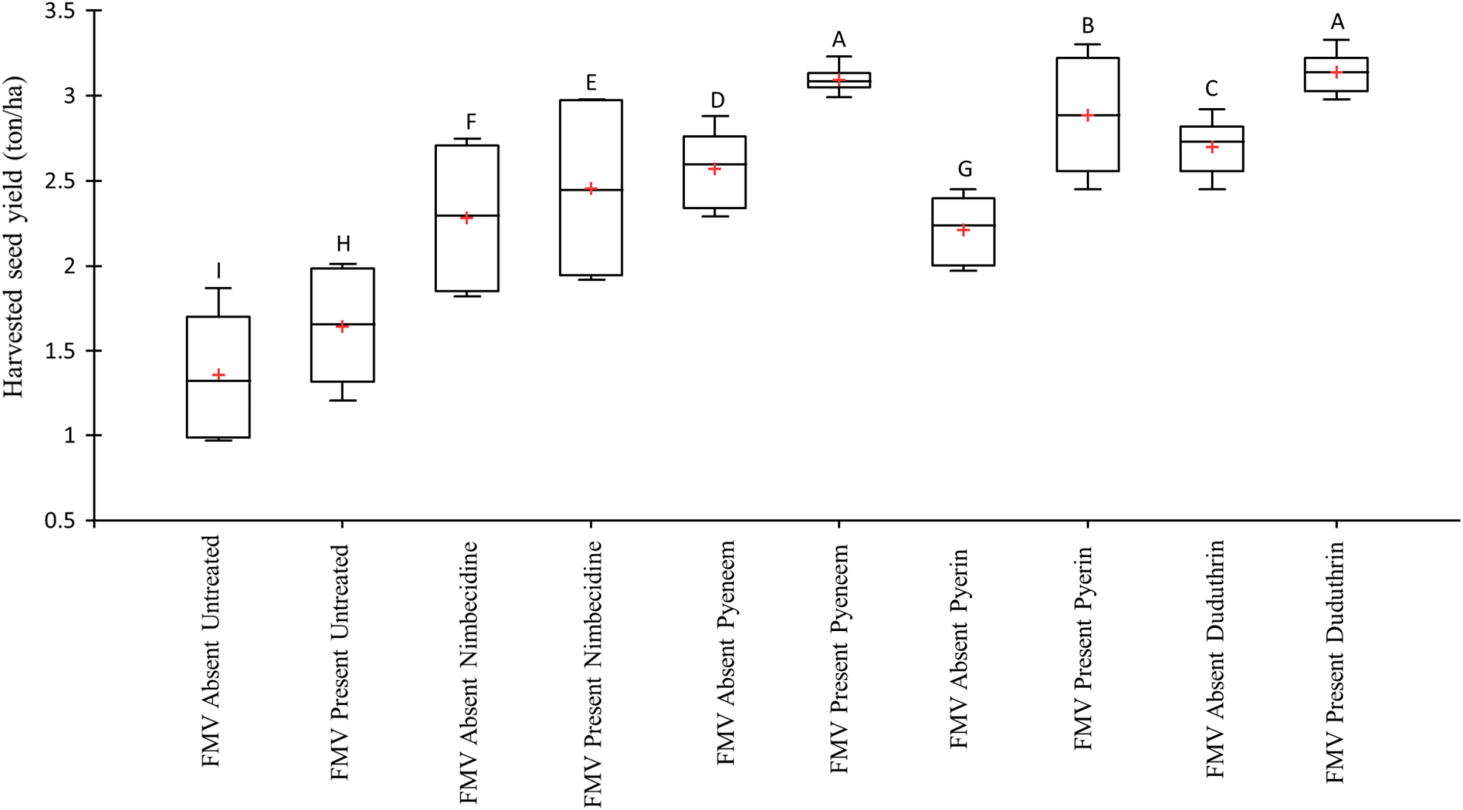
Lablab bean yield from botanical insecticides (Nimbecidine, Pyeneem and Pyerin), Duduthrin (Lambdacyhalothrin) and untreated as positive and negative controls, respectively, in the presence or absence of field margin (FMV). Letters above each box plot are from a post‐hoc Least Significant Difference test showing differences in mean values at the 95% confidence interval [Colour figure can be viewed at wileyonlinelibrary.com]

The presence of field margin vegetation enhanced the yield for each crop protection method employed (Figure 4). The lowest yield was observed in the untreated control. The highest yields were achieved when treating the crop with the botanical insecticide Pyeneem and the synthetic Duduthrin in the presence of field margin vegetation. The next best treatment was Pyerin with field margin present, thereafter, followed by the treatments without field margins and Nimbecidine. Nimbecidine and the untreated control were observed to have relatively high variability in yields compared with the other treatments. An Analysis of Variance on all the yield parameters collected at the time of harvest (plant height, undamaged pods, damaged pods, seeds per pod, 100 seed weight, grain yield, crop plant biomass) showed consistent effects of the treatments on crop production (Appendix S2).

## 4 DISCUSSION

This study demonstrated the potential of integrating biorational pest management options by combining botanical insecticides and field margin vegetation to support agroecological intensification and sustainable management of aphid pests in the orphan crop legume Lablab. Our data showed that the use of botanical insecticides can deliver similar Lablab bean yields as those achieved with synthetic pesticides but with reduced impact on natural enemies of pests. This is consistent with other related studies undertaken by Tembo et al. (2018), Campos et al. (2019) and Soares et al. (2019). Furthermore, the abundance of natural enemies that contribute to biorational pest management can be enhanced by florally rich margins around the crop that provide food and refuge for natural enemies that later move into crop fields for biological control and a potential buffer against migrating pests (Bianchi & Wäckers, 2008; Knapp & Řezáč, 2015; Quispe et al., 2017; Skirvin et al., 2011).

Generally, lower aphid abundance, damage severity and percent incidence were observed in plots with florally rich margins. The combination of botanical insecticides and field margins resulted in significantly reduced bean aphid infestation compared with applying the insecticides in plots without field margins demonstrating that co‐opting multiple agroecological approaches can deliver pest management outcomes that are as effective or even more so than relying on synthetic insecticides. Our data are consistent with Amoabeng et al. (2020) who reported high insect pest suppression when botanical insecticides and habitat manipulation were integrated. Non‐host plants can, however, reduce an insect herbivores’ capacity to locate and colonize host plants through chemical and physical interference (Mansion‐Vaquié et al., 2020) and this may also have contributed to the outcomes recorded here.

The application of the synthetic insecticide, Duduthrin (Lambdacyhalothrin 17.5 g/l), was the most effective treatment for reducing aphid infestation. This was expected considering that it is a broad‐spectrum insecticide that is used widely in managing insect pests and registered for use on a range of crops (Belmain et al., 2013). The botanical insecticides evaluated here have also been demonstrated to be effective in the management of insect pests though not previously evaluated alongside crop margin flowers (Saleem et al., 2019). The active ingredients; pyrethrins in pyrethrum and terpenoids such as azadirachtin in neem‐based insecticides, are known to be effective against aphids with repellent and antifeedant activity, and growth and reproduction inhibition against a range of other pests arthropods (Pezzini & Koch, 2015; Ulrichs et al., 2001) and notably against aphids and other hemipterans on other legume crops (Fite et al., 2020; Nahashon et al., 2016; Pezzini & Koch, 2015). However, variable efficacy of botanical insecticides on insect pests has been reported. This loss of efficacy is partly attributed to differences in their mode of action and the capacity of pests to detoxify the active ingredients (Sisay et al., 2019). In addition, the active ingredients of pyrethrum and neem are labile in ultraviolet light. However, this also means they are non‐persistent and thus more compatible with conservation biological control as the compounds are less likely to harm beneficial insects (Soares et al., 2019). This loss of efficacy presents a challenge to the adoption of botanical insecticides. This may be overcome by combining their use with enriched agricultural landscapes as demonstrated here with our data, which shows that enriched margins around crops can enhance populations of natural enemies even in combination with botanical insecticide applications.

Nimbecidine was generally the least effective botanical insecticide in reducing aphid infestation but had comparable effects on natural enemy insect numbers to Pyerin and Pyeneem. Although Pyerin and Pyeneem were generally as effective in reducing aphid infestations as the synthetic Duduthrin, these plots showed a higher abundance of natural enemies post‐spray. Duduthrin‐treated plots had the lowest natural enemy abundance, and this was especially severe in plots not surrounded by non‐crop margin flowers. The low abundance of natural enemies was likely due to the high entomotoxicity of lambda‐cyhalothrin, the active ingredient in Duduthrin, which suppresses populations of both insect pests and their natural enemies (Mkenda et al., 2015; Mkindi et al., 2017).

The compatibility of botanical insecticides with other IPM approaches is not in itself new and has been proposed and reported previously; for example, with entomopathogenic fungi and natural enemies of pests (Fernandez‐Grandon et al., 2020). Field margin vegetation has also recently been demonstrated to be complimentary to conservation biological control as the margin plants offer alternative food resources (Mkenda et al., 2019) and illustrates the potential synergies and compatibilities of integrating botanical insecticides and enhanced non‐crop habitats for improved insect pest suppression (Arnold et al., 2021; El‐Wakeil, 2014). Such compatibility was demonstrated by Amoabeng et al. (2020) who evaluated the dual pest management services of botanical insecticides and conservation biological control for managing brassicas pests and along with our data further support the scope for combining direct pest management interventions with enhanced landscapes that support natural pest regulating processes. In particular, this may enhance the recovery of natural enemy populations after exposure to synthetic and botanical insecticide applications.

The mortality and recovery of insects after exposure to botanical insecticide active ingredients have been shown to vary across insect families. Khan et al. (2015) reported low adult mortality of six‐spotted ladybird beetles (*Menochilus sexmaculatus* Fab.) (Coccinellidae) when exposed to neem oil. Similarly, lacewings (Chrysopidae) have been shown to have a high tolerance to pyrethrins due to increased levels of pyrethroid esterase (Amarasekare & Shearer, 2013). El‐Wakeil et al. (2006) reported no mortality of lacewings due to neem‐based pesticides like NSE 5%, Neemark, Achook and Nimbecidine each at 0.003%. Studies on Hymenoptera parasitoids have shown variable outcomes after exposure to botanical insecticides. High mortality on adult parasitoids, decreased parasitism and reduced parasitoid emergence after exposure to neem‐based insecticides have been demonstrated (Monsreal‐Ceballos et al., 2018). However, the egg parasitoid *Trichogramma pretiosum* showed low mortality when treated with azadirachtin (Almeida et al., 2010). The difference in parasitoid responses to botanical insecticides has been attributed to factors such as active ingredients, type of exposure, parasitoid species and stage of development (Monsreal‐Ceballos et al., 2018). The application of botanical insecticides may enhance the conservation of natural enemies owing to the reduced mortality compared with those exposed to synthetic applications and therefore, may contribute to the success of integrated pest management (IPM) programs (Mkenda et al., 2015). In particular, the integration of botanical insecticides with flower‐rich field margin provided additional benefits in the conservation of natural enemies and insect pest suppression complimenting other recent studies (Amoabeng et al., 2020). However, precautions should be taken to ensure that the botanical insecticides are applied at the recommended rates since high rates have been reported to cause higher mortality rates of beneficial insects (Pezzini & Koch, 2015).

Bean aphids have been shown to have a significant effect on the grain yield as they directly affect the photosynthetic ability of the leaves. A related study by Mwangi et al. (2008) reported significant grain yield reduction in susceptible common bean (*Phaseolus vulgaris*) varieties to *Aphis fabae*. The results from this study indicated that flower‐rich field margins could increase grain yield. In addition, the combination of field margins and botanical insecticides resulted in higher grain yield compared with the use of botanical insecticides in absence of plot margin flowers. The impact of the three botanical insecticides on natural enemy populations was generally similar, but the lower yield achieved with Nimbecidine in comparison with Pyerin or Pyeneem suggests the latter are more suitable for IPM on Lablab.

This study demonstrates that commercial botanical insecticides have reduced impacts on key natural enemies of aphids compared with synthetics, in combination with florally enhanced landscapes and illustrate the compatibility of approaches, supporting the concepts of IPM in sustainable cropping systems and conservation biological control. Using botanical insecticides alongside field margin management for flowering plants provides a sustainable pest management approach that is environmentally benign compared with synthetic insecticides along with corresponding higher grain yield.

## AUTHOR CONTRIBUTION

PCS, SRB, JOO and SEJA conceived the study. JOO, PKB, JGN, EKC, RMSM, SEJA, SRB and PCS were involved in the study design. LOO and SRB carried out the statistical analysis, LOO wrote the first draft of the manuscript. LOO carried out the field trials and data collection. All authors were involved in writing the manuscript and gave final approval for publication

## CONFLICT OF INTEREST

The authors declare that the research was conducted in the absence of any commercial or financial relationships that could be construed as a potential conflict of interest.

## Supporting information


Appendix S1

Appendix S2

## Data Availability

The data supporting the findings of this study are available from https://zenodo.org/record/5132875#.YPxWvkxRXIU. Data Citation: Lawrence Ochieng, Joshua Ogendo, Philip Bett, Jane Nyaanga, Erick Cheruiyot, Richard Mulwa, Sarah Arnold, Steven Belmain & Philip Stevenson (2021). Botanicals for ecomanagement of aphids [Data set]. *Journal of Applied Entomology*. Zenodo. http://doi.org/10.5281/zenodo.5132875
