# Supplementary figures and images for: Field margins and botanical insecticides enhance Lablab purpureus yield by reducing aphid pests and supporting natural enemies
Source: J Appl Entomol. 2022 May 20;146(7):838–49. doi: 10.1111/jen.13023 (PMC9545213; doi:10.1111/jen.13023)

**Online resource 1:** Field experiment layout

**
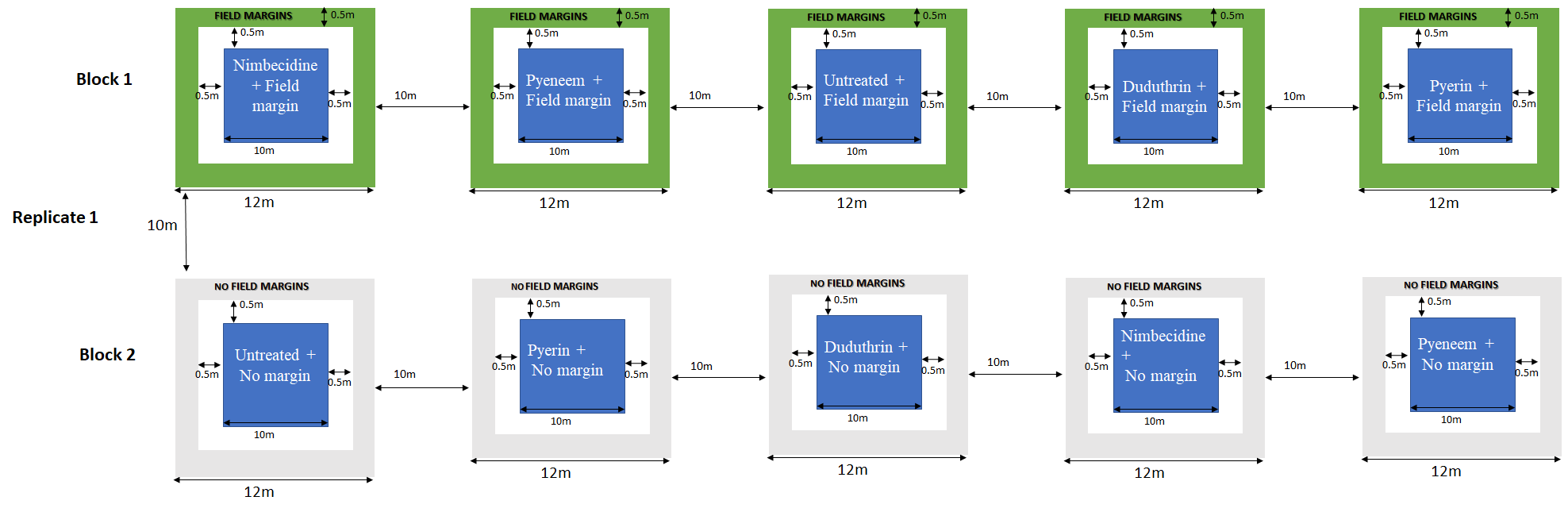
**

Supplement: Supplementary file 1 — Appendix S1 [file JEN-146-838-s002.docx]

**Online resource 2:** Field experiment layout

**
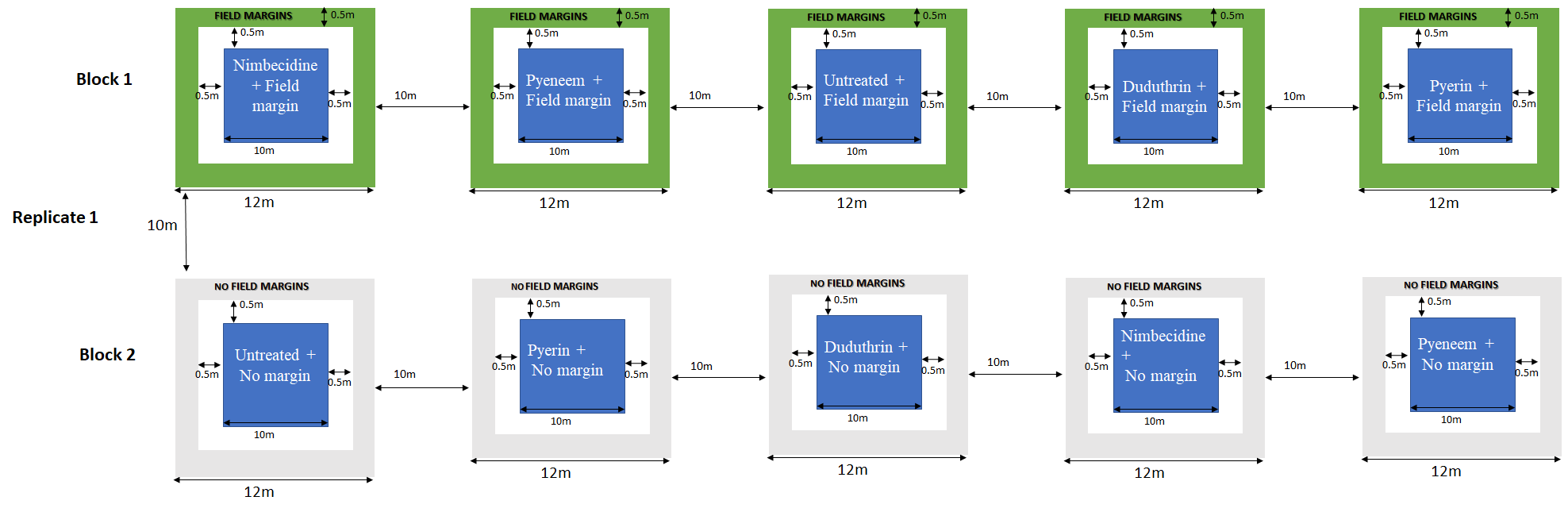
**

Supplement: Supplementary file 2 — Appendix S2 [file JEN-146-838-s001.docx]
